# Si Nanocrystal-Embedded SiO_*x*_ nanofoils: Two-Dimensional Nanotechnology-Enabled High Performance Li Storage Materials

**DOI:** 10.1038/s41598-018-25159-4

**Published:** 2018-05-02

**Authors:** Hyundong Yoo, Eunjun Park, Juhye Bae, Jaewoo Lee, Dong Jae Chung, Yong Nam Jo, Min-Sik Park, Jung Ho Kim, Shi Xue Dou, Young-Jun Kim, Hansu Kim

**Affiliations:** 1 Department of Energy Engineering, Hanyang University, 222 Wangsimni-ro, Seongdong-gu Seoul, 133–791 Republic of Korea; 2 Institute for Superconducting and Electronic Materials (ISEM), Australian Institute for Innovative Materials (AIIM), University of Wollongong, North Wollongong, New South Wales 2500 Australia; 3 Advanced Batteries Research Center, Korea Electronics Technology Institute, Seongnam, 463-816 Republic of Korea; 4 Department of Advanced Materials Engineering for Information and Electronics, Kyung Hee University, Yongin, 17104 Republic of Korea; 5 SKKU Advanced Institute of Nanotechnology (SAINT), Sungkyunkwan University, Suwon, 16419 Republic of Korea

## Abstract

Silicon (Si) based materials are highly desirable to replace currently used graphite anode for lithium ion batteries. Nevertheless, its usage is still a big challenge due to poor battery performance and scale-up issue. In addition, two-dimensional (2D) architectures, which remain unresolved so far, would give them more interesting and unexpected properties. Herein, we report a facile, cost-effective, and scalable approach to synthesize Si nanocrystals embedded 2D SiO_*x*_ nanofoils for next-generation lithium ion batteries through a solution-evaporation-induced interfacial sol-gel reaction of hydrogen silsesquioxane (HSiO_1.5_, HSQ). The unique nature of the thus-prepared centimeter scale 2D nanofoil with a large surface area enables ultrafast Li^+^ insertion and extraction, with a reversible capacity of more than 650 mAh g^−1^, even at a high current density of 50 C (50 A g^−1^). Moreover, the 2D nanostructured Si/SiO_*x*_ nanofoils show excellent cycling performance up to 200 cycles and maintain their initial dimensional stability. This superior performance stems from the peculiar nanoarchitecture of 2D Si/SiO_*x*_ nanofoils, which provides short diffusion paths for lithium ions and abundant free space to effectively accommodate the huge volume changes of Si during cycling.

## Introduction

Graphene, a typical 2D allotrope of carbon, has aroused great attention as one of the most exciting materials for numerous applications because of its distinctive properties^1,2^. Various 2D nanostructured materials, including metal oxides, metal sulfides, and metal nitrides, have been widely studied for practical use in energy storage applications owing to their impressive characteristics^2–6^. We anticipate that configuration of 2D nanofoils would provide a great opportunity to greatly improve the electrochemical performance of various Li^+^ storage materials, in particular, Si-based anode materials, that suffer from large volume variation and undesirable conductivity loss induced by mechanical degradation of Si during cycling^7–9^. The unique morphology of 2D nanostructured Si-based materials would facilitate fast Li^+^ insertion and extraction by enlarging the active surface area and shortening the diffusion length for Li^+^ ^2–5,10^. Furthermore, such a 2D nanostructure represents one of the most promising material architectures to effectively relax the mechanical strain induced by the large volume variation of Si during cycling^11–20^.

Despite these outstanding advantages of 2D morphology, the synthesis of 2D nanostructured materials has been very limited and applied to only a few materials *via* chemical vapor deposition (CVD) or self-assembly^21–30^. To the best of our knowledge, indeed, there are few reports on Si-based 2D nanostructured materials and their applications so far^31–33^. In particular, the currently available processes are not suitable for the synthesis of Si-based nanofoils because of high production costs and time-consuming processes^10,26–33^. Recently, we have developed a highly reliable Si/SiO_*x*_ nanocomposite *via* a sol-gel reaction, which exhibited a high reversible capacity (>800 mAh g^−1^) and excellent cycling performance^34–36^. In a continuing effort to exploit advanced Si/SiO_*x*_ nanocomposite using this synthetic approach, we here propose a facile and scalable synthesis of large-area Si/SiO_*x*_ 2D nanofoils with a thickness of about 8 nm as a potential Li^+^ storage material for lithium ion batteries (LIBs) *via* a solution evaporation induced interfacial sol-gel reaction. We expect that unique 2D Si/SiO_*x*_ nanofoils can effectively minimize the inevitable mechanical strain induced by Li^+^ insertion, thereby promoting strong enhancement in Li^+^ storage capability. More importantly, our newly developed approach presented here allows the synthesis of facile, cost-effective, and scalable 2D Si/SiO_*x*_ nanofoils in an aqueous solution by promoting lateral growth of hydrogen silsesquioxane (HSiO_1.5_, HSQ) nanofoils at the interface between water and air.

## Results

### Design and synthesis of 2D Si/SiO_x_ nanofoils

**Figure 1:**
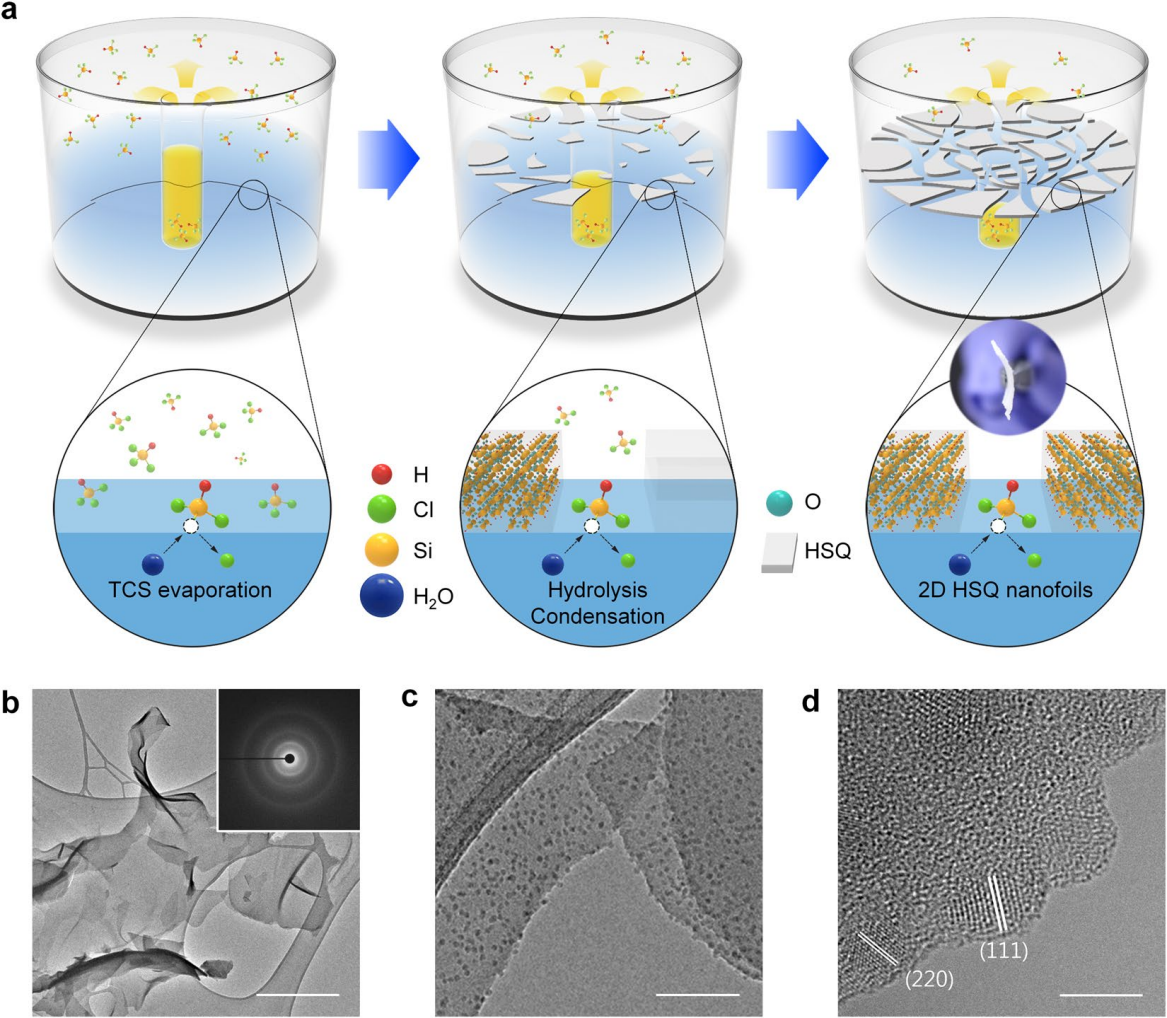
Schematic illustration of 2D HSQ nanofoil preparation process and structural characterization of 2D Si/SiO_*x*_ nanofoils. (**a**) 2D HSQ nanofoils synthesized using sol-gel reaction in a home-made reactor, and 2D Si/SiO_*x*_ nanofoils after heat treatment of as-prepared 2D HSQ nanofoils at 1000 °C; (**b**) low-magnification TEM image with a corresponding selected area diffraction pattern (SADP) (inset), (**c**) high-magnification TEM image, and (**d**) HRTEM image of 2D Si/SiO_*x*_ nanofoils. Scale bar, (**b**) 1 μm, (**c**) 100 nm, and (**d**) 5 nm.

Fig. 1 shows the preparation of 2D Si/SiO_*x*_ nanofoils *via* a solution evaporation-induced interfacial sol-gel reaction to form laterally grown 2D HSQ nanofoils with transmission electron microscopy (TEM) images. The process was deliberately designed after careful consideration of various synthetic parameters, such as the vapor pressure of precursor and the reaction temperature. Considering the high vapor pressure of trichlorosilane (HSiCl_3_) precursor at room temperature (Fig. S1)^37,38^, a sealed home-made reactor composed of a precursor container and a water reservoir was carefully designed, as schematically illustrated in Fig. 1a. HSiCl_3_ vapor is spontaneously transported into the water (left image of Fig. 1a) at room temperature under ambient pressure. The formation of HSQ nanofoils can be initiated at the interface between water and air in a reactor (center image of Fig. 1a) through a sol-gel reaction of HSiCl_3_ with the help of water. The lateral growth of 2D HSQ nanofoils proceeds spontaneously until reaching atmospheric equilibrium in the reactor (right image of Fig. 1a).

The resulting 2D HSQ nanofoils with an amorphous structure (Fig. 1b and Fig. S2) were heated at 1000 °C for 1 h under Ar atmosphere, and finally Si nanocrystals embedded SiO_*x*_ nanofoils were successfully obtained (Fig. 1c). We confirmed the lateral growth of the 2D Si/SiO_*x*_ nanofoils, in which the Si nanocrystals have a typical crystalline structure with a size of about 5 nm. From a magnified TEM image (Fig. 1d), the *d*-spacing of Si nanocrystals embedded in amorphous SiO_*x*_ matrix was measured to be 0.311 and 0.118 nm, corresponding to the interlayer distances of the (111) and (220) planes of crystalline Si, respectively^34,35,39–43^. Elemental mapping using TEM tomography also revealed that the Si/SiO_*x*_ nanofoils are mainly composed of Si and O, where Si-rich phase presumed to be Si nanocrystals is also evident (Fig. S3). What is important thing is that the synthetic route proposed in this study for obtaining 2D Si/SiO_*x*_ nanofoils is a scalable and cost-effective process, as the synthetic process does not need either an ultrahigh vacuum with expensive facilities or high pressure for ensuring the correct hydrothermal environment.

**Figure 2:**
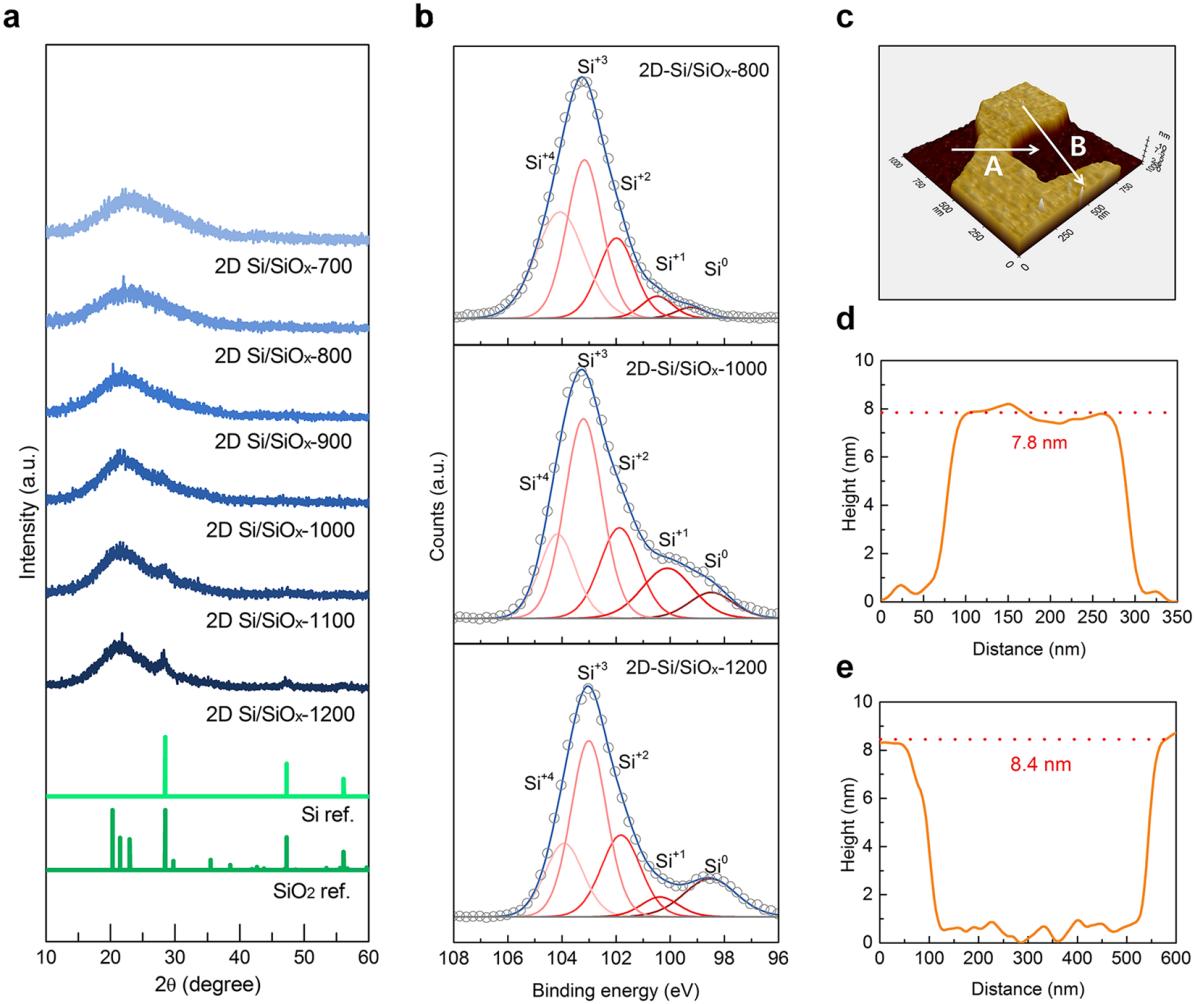
(**a**) XRD patterns of 2D Si/SiO_*x*_ nanofoils, (**b**) Si 2p XPS spectra of 2D Si/SiO_*x*_ nanofoils prepared at different temperatures (800 °C, 1000 °C, 1200 °C), and (**c**–**e**) thickness of 2D Si/SiO_*x*_ nanofoils along line with AFM image of 2D Si/SiO_*x*_ nanofoils prepared at 1000 °C; (**d**) line A, and (**e**) line B.

Considering the strong correlation between the electrochemical performance and the microstructure of Si/SiO_*x*_ nanofoils, the structural evolution of the 2D Si/SiO_*x*_ nanofoils was further investigated by heating the as-prepared 2D HSQ nanofoils at various temperatures ranging from 700 to 1200 °C. Fig. 2a shows powder X-ray diffraction (XRD) patterns of 2D Si/SiO_*x*_ nanofoils synthesized at different temperatures. We found a broad peak at a low Bragg angle in all samples, which is a typical characteristic of amorphous SiO_*x*_ phase^42^. Interestingly, an additional Bragg peak appeared at 28.4° after heating the sample above 1000 °C, which corresponds to the (111) planes of crystalline Si^34,43^ and it was gradually grown by increasing the temperature up to 1200 °C. Further inspection of the 2D Si/SiO_*x*_ nanofoils was carried out by X-ray photoelectron spectroscopy (XPS) and atomic force microscopy (AFM) and Brunauer, Emmett and Teller(BET) method. According to the Si 2p XPS spectra of 2D Si/SiO_*x*_ nanofoils prepared at different temperatures (Fig. 2b), we found that Si^3^^+^ species (102.8 eV) is a main component in the Si/SiO_*x*_ nanofoils. Importantly, the growth of elemental Si (Si^0^) was observed at 98.4 eV with increasing the synthesis temperature. This clearly reveals the further reduction and coarsening of Si nanocrystals embedded in SiO_*x*_ matrix. The corresponding O 1 s spectra also supports continuous loss of binding of Si with O with increasing heating temperature, as evidenced by the growth of the Si-O peak at 531.9 eV (Fig. S4)^42–44^. From AFM observations (Fig. 2c–e), we also confirmed that ultrathin 2D Si/SiO_*x*_ nanofoils with a thickness of ~8 nm were successfully synthesized over a large area by the proposed interfacial sol-gel reaction of trichlorosilane. From the N_2_ adsorption and desorption isotherm curves of 2D Si/SiO_*x*_ and Si/SiO_*x*_ nanosphere (Fig. S5), we note that 2D Si/SiO_*x*_ has larger surface area of 53.0 m^2^g^−1^ than that of Si/SiO_*x*_ nanophere (13.2 m^2^g^−1^) thanks to its 2D morphology.

**Figure 3:**
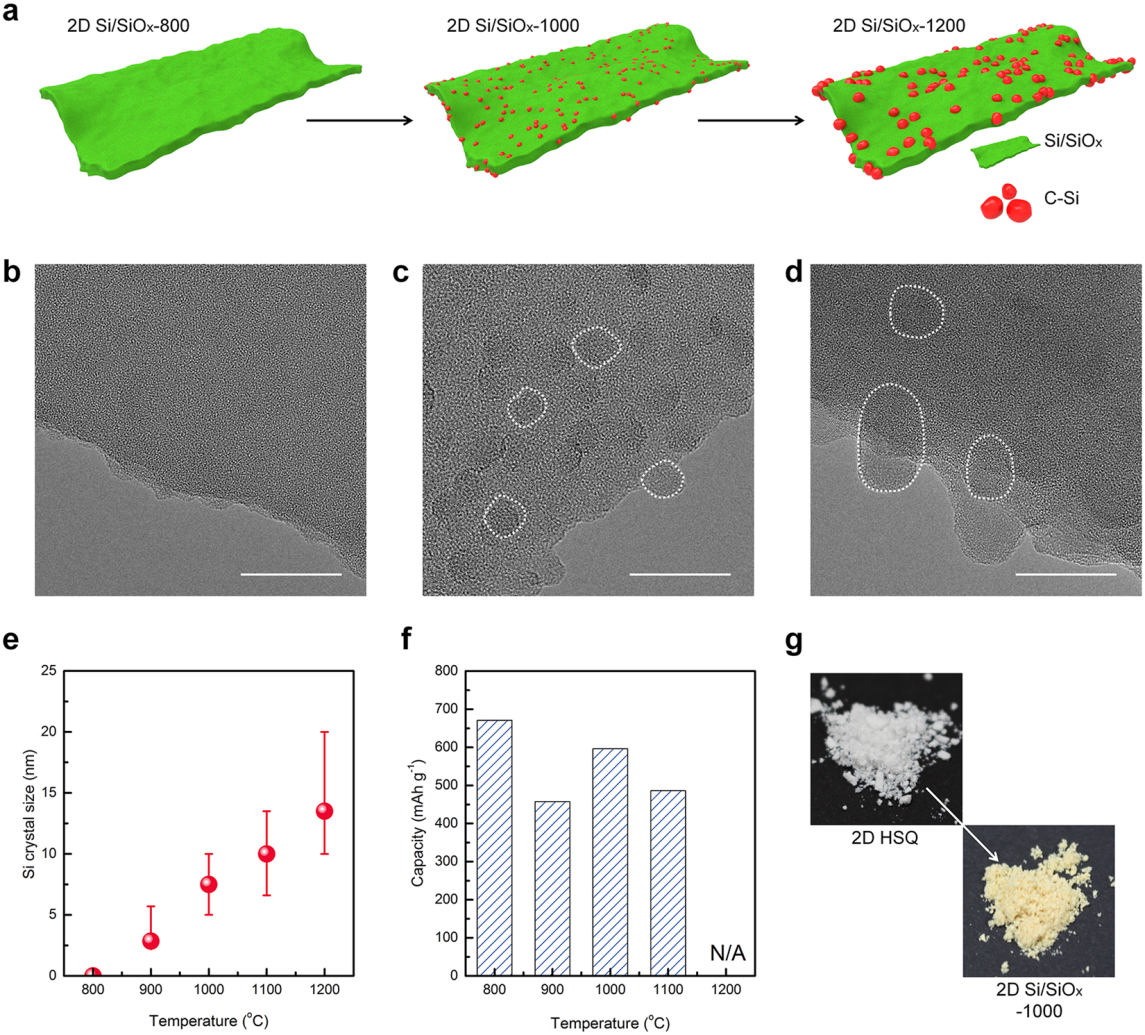
Structural characterization of 2D Si/SiO_*x*_ nanofoils obtained by heat treatment at various temperatures. (**a**) Schematic diagram of various 2D Si/SiO_*x*_ nanofoils, HRTEM images of 2D Si/SiO_*x*_ nanofoils; (**b**) 800 °C, (**c**) 1000 °C, (**d**) 1200 °C, and comparisons of (**e**) Si crystal size, (**f**) discharge capacity of 2D Si/SiO_*x*_ nanofoils, and (**g**) digital images of 2D Si/SiO_*x*_ nanofoils before and after heat treatment. Scale bar, (**b**–**d**) 20 nm.

Consistently, a gradual growth of the Si nanocrystals can be identified as described in Fig. 3a. The corresponding TEM results confirmed that there was no evidence for Si nanocrystals in the amorphous SiO_*x*_ matrix after heating temperature as low as 800 °C (Fig. 3b), while the growth of Si nanocrystals was clearly observed at higher heating temperatures above 1000 °C (Fig. 3c,d). The size of Si nanocrystals embedded in the SiO_*x*_ nanofoils prepared at 1000 °C was measured to be approximately 7.5 nm, and larger Si nanocrystals (about 13.5 nm) were obviously found in the Si/SiO_*x*_ nanofoils prepared at 1100 °C, respectively. The crystallite size of Si embedded in amorphous SiO_*x*_ matrix was estimated using the Scherrer equation (Fig. 3e)^45,46^, which is well matched with the TEM observations. It is worth noting that the formation of Si nanocrystals is accelerated by increasing the heating temperature above 1000 °C, implying that the relatively high heating temperature could facilitate the reduction of HSQ into Si.

On the other hand, the chemical composition and configuration of 2D Si/SiO_*x*_ nanofoils would be predominant factors for determining their electrochemical performance. Since large-sized Si nanocrystals would have a negative effect on the electrochemical performance of the 2D Si/SiO_*x*_ nanofoils, the optimum size for the Si nanocrystals embedded in amorphous SiO_*x*_ should be found in advance by varying the heat-treatment temperature. Bearing this in mind, we systematically compared the electrochemical behavior of 2D Si/SiO_*x*_ nanofoils prepared at different temperatures. There was a strong correlation between the initial specific capacity and the size of Si nanocrystals embedded in the 2D Si/SiO_*x*_ nanofoils, as shown in Fig. 3f. We found that the optimum temperature for the synthesis of 2D Si/SiO_*x*_ nanofoils is 1000 °C in view of the achieved specific capacity and cycling stbaility. After heating at 1000 °C the white-colored 2D HSQ nanofoils changed to brown-colored 2D Si/SiO_*x*_ nanofoils by forming abundant Si nanocrystals in the amorphous SiO_*x*_ matrix (Fig. 3g).

### Electrochemical performance of 2D Si/SiO_x_ nanofoils

**Figure 4:**
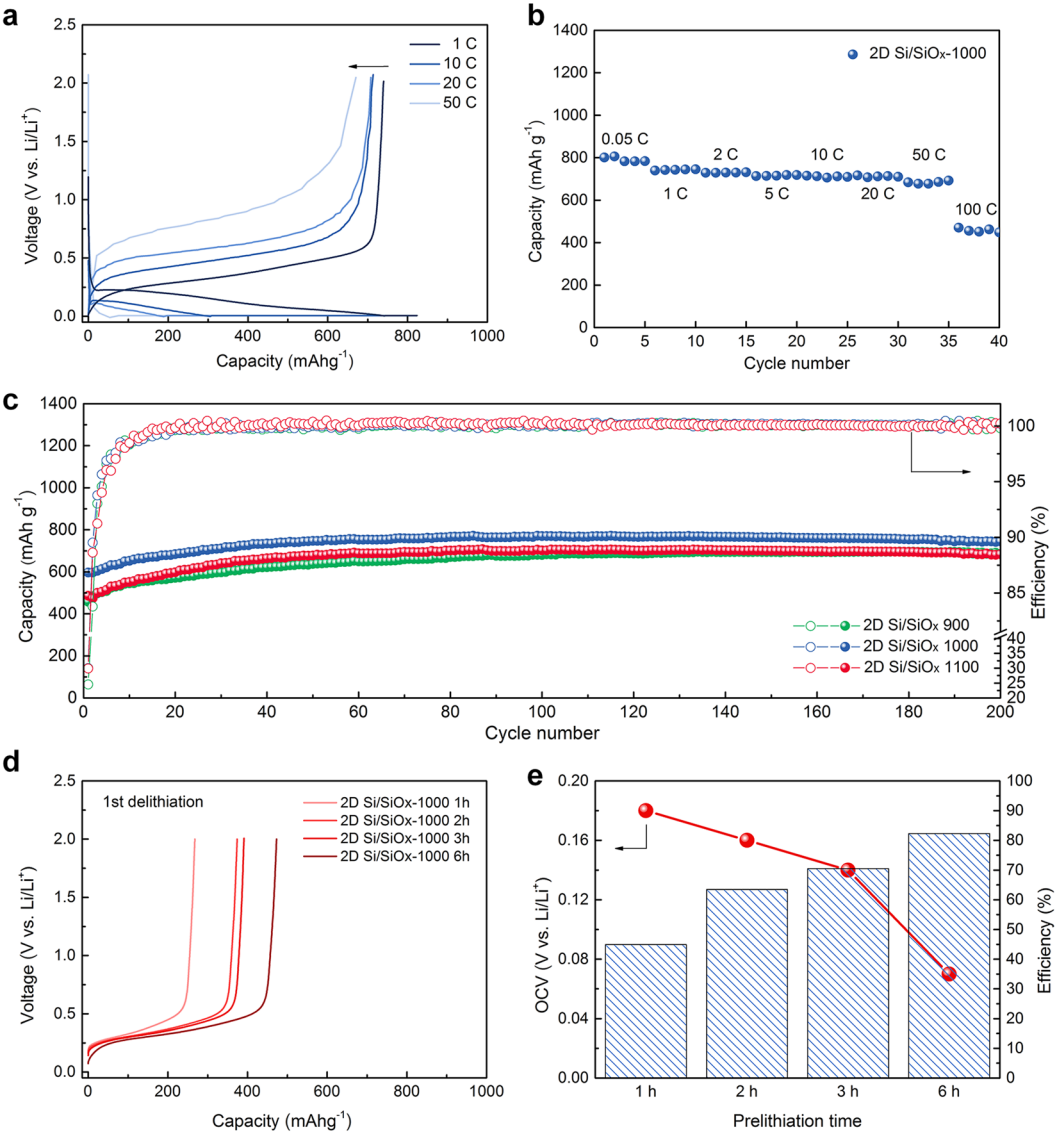
Electrochemical performance of 2D Si/SiO_*x*_ nanofoils. (**a**) Galvanostatic voltage profiles of 2D Si/SiO_*x*_ nanofoils (1000 °C) collected at different current densities of 1, 10, 20, and 50 C, (**b**) rate-capability of a Si/SiO_*x*_ nanofoil (1000 °C) anode at different current densities of 0.05, 1, 2, 5, 10, 20, 50, and 100 C (1 C = 1000 mA g^−1^), (**c**) cycling performance of 2D Si/SiO_*x*_ nanofoil electrodes for samples prepared at different temperatures at a constant current of 0.2 C (200 mA g^−1^) for 200 cycles, (**d**) discharge (Li^+^ extraction) profiles of pre-lithiated 2D Si/SiO_*x*_ nanofoil (1000 °C) electrodes at a constant current of 0.2 C (200 mA g^−1^)

The electrochemical Li^+^ storage behaviors of the 2D Si/SiO_*x*_ nanofoils was thoroughly examined by galvanostatic charge (Li^+^ insertion) and discharge (Li^+^ extraction) tests at various current densities as shown in Fig. 4a,b. The reversible capacity of the 2D Si/SiO_*x*_ nanofoils synthesized at 1000 °C was estimated to be 739.8 mAh g^−1^ at a current density of 1 A g^−1^ (1 C), and 90.6% of their reversible capacity can be retained, even at ultra-high current density of 50 A g^−1^ (50 C), indicating that about 90% of the capacity can be charged within 72 seconds. This impressive rate capability of the 2D Si/SiO_*x*_ nanofoils is mainly attributable to the large surface area, as well as the short Li^+^ diffusion paths allowed by the 2D nanoarchitecture, which are advantageous for enhancing the electrochemical kinetics of Li^+^ mobility during cycling. The resulting ultra-fast charging characteristics could encourage the potential use of 2D Si/SiO_*x*_ nanofoils in high-power LIB applications.

Another promising feature of the 2D Si/SiO_*x*_ nanofoils is their excellent cycle performance towards repeated Li^+^ insertion and extraction. Fig. 4c shows the cycling performance of the 2D Si/SiO_*x*_ nanofoils synthesized at 900, 1000, and 1100 °C. All the samples showed a gradual increase in their reversible capacities in the first few cycles, and then the reversible capacities reached a steady state with repeated cycling. Such behavior has been generally observed in SiO_*x*_ based anode materials due to the activation of amorphous SiO_*x*_ matrix towards Li^+^ insertion and extraction^34–36,47^. We would like to emphasize that the Coulombic efficiencies of the 2D Si/SiO_*x*_ nanofoils reached more than 99.5% within the first few cycles, which is comparable to the performance of commercial graphite anode for current LIBs^48–50^. The 2D Si/SiO_*x*_ nanofoils prepared at 1000 °C still maintained a high reversible capacity of 744.3 mAh g^−1^, even after 200 cycles, without significant capacity fading. We believe that the excellent cycling performance is mainly attributable to the favorable morphology and microstructure of the 2D Si/SiO_*x*_ nanofoils. In contrast, low-temperature synthesized 2D Si/SiO_*x*_ (i.e. 700 and 800 °C) showed relatively low reversible capacities during cycling because Si nanocrystals could not be properly formed in SiO_*x*_ matrix (Fig. S5).

On the other hand, the low initial Coulombic efficiency of Si-based anode materials is regarded as a major drawback causing a significant energy loss of LIBs. Despite its great advantages, the proposed 2D Si/SiO_*x*_ nanofoil anode also exhibited a low initial Coulombic efficiency (~32.8%). Thus, a fundamental solution should be addressed to make it viable for practical use. In this respect, we demonstrate a pre-lithiation of the 2D Si/SiO_*x*_ nanofoil anode to improve its initial Coulombic efficiency without any performance fading. As shown in Fig. 4d, the discharge capacity is dependent on the pre-lithiation time and the initial Coulombic efficiency of the 2D Si/SiO_*x*_ nanofoil anode could be increased more than 82% after the pre-lithiation for 6 h (Fig. 4e). We believe that further improvement of the initial Coulombic efficiency can be expected by the optimization of pre-lithiation parameters.

In addition to the initial Coulombic efficiency, one of the most critical requirements for Si-based materials is to ensure thermal stability, even under high temperature operation. It is well recognized that an increased active surface area can decrease thermal stability of the electrode material in LIBs, it is very much required to investigate the thermal stability of nanostructured materials for their practical use. To this end, the cycling performance of the proposed 2D Si/SiO_*x*_ nanofoil anode was evaluated at the high temperature of 60 °C. Note that there was no significant performance fading over 100 cycles, even at the elevated temperature (Fig. S6). Given that the long-term stability and thermal stability of Si-based anode materials are crucial for meeting the rigorous standards for commercial use for LIBs, these as-prepared 2D Si/SiO_*x*_ nanofoils would be a solution for building advanced LIBs to meet the difficult requirements of emerging LIB applications such as electric vehicles and large-scale energy storage systems for grid support.

### Dimensional stability of 2D Si/SiO_x_ nanofoils

**Figure 5:**
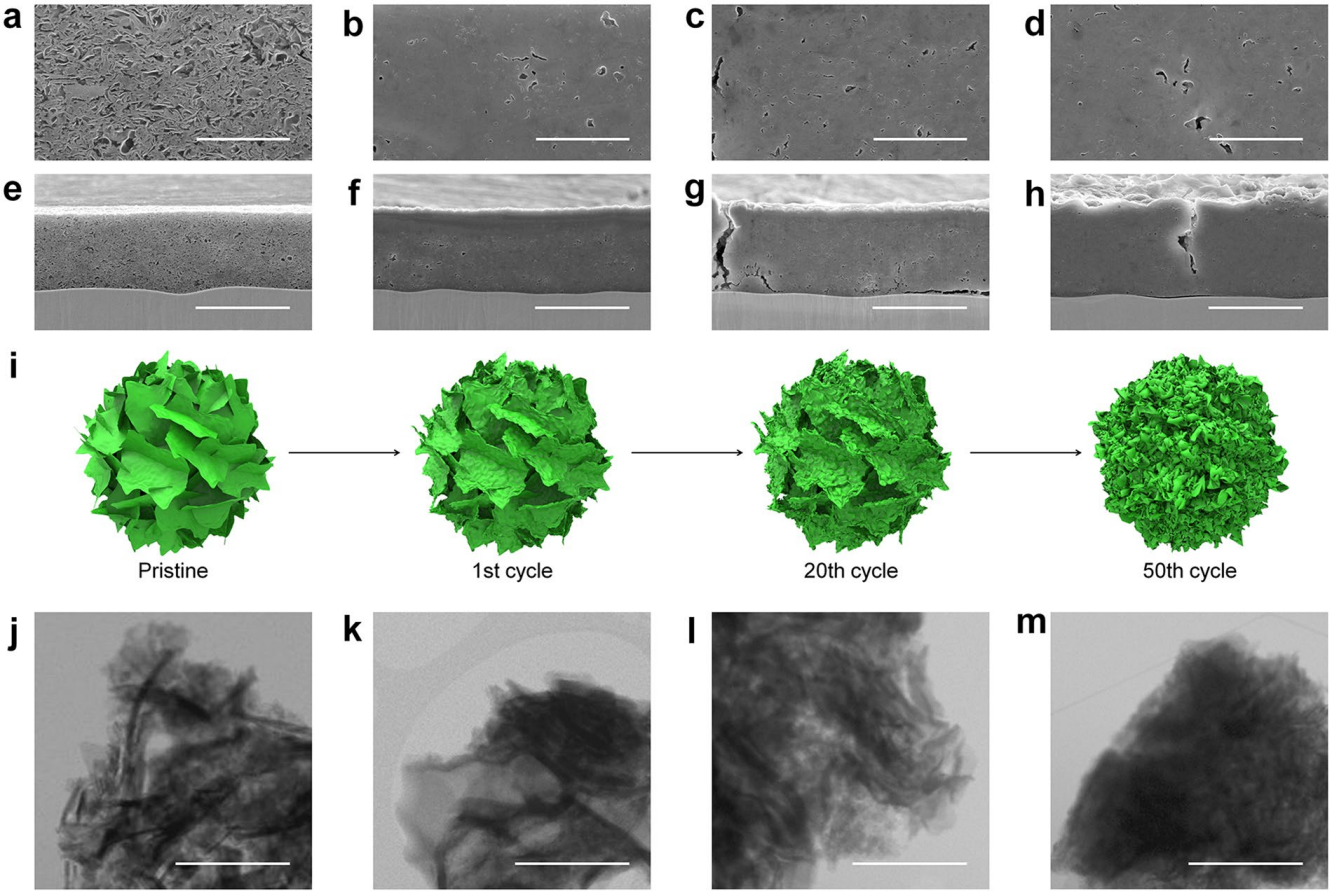
Comparison of structural stability of 2D Si/SiO_*x*_ nanofoils (1000 °C) collected at different cycles. Top view FESEM images (**a**–**d**), cross-sectional FESEM images (**e**–**h**), and TEM images (j-m) of 2D Si/SiO_*x*_ (1000 °C) electrode: pristine (**a**,**e**,**j**), after 1 cycle (**b**,**f**,**k**), after 20 cycles (**c**,**g**,**l**), and after 50 cycles (**d**,**h**,**m**), and (**i**) schematic illustrations of 2D Si/SiO_*x*_ nanofoils at different cycles. Scale bar, (**a**-**d**) 5 μm, (**e**–**h**) 20 μm, and (**j**–**m**) 1 μm.

It is argued that the excellent dimensional stability of 2D Si/SiO_*x*_ nanofoil electrode is another noteworthy advantage characteristic of 2D nanostructured materials. Fig. 5a shows field-emission scanning electron microscopy (FESEM) images of pristine Si/SiO_*x*_ nanofoil electrode, in which abundant nanopores were effectively created by the unique 2D morphology of the Si/SiO_*x*_ nanofoils. The resulting nanosized free space would play a critical role in accommodating the volume expansion of Si/SiO_*x*_ nanofoil electrode induced by Li^+^ insertion.

Compared with pristine 2D Si/SiO_*x*_ nanofoil electrode with a thickness of 15.76 μm (Fig. 5e), the electrode was expanded by less than 8% (17.04 μm) of its initial thickness after the first cycle (Fig. 5f). Moreover, the thickness expansion of the electrode was only 18% (18.68 μm) of its initial thickness after 20 cycles (Fig. 5g), and 28% (20.22 μm) even after 50 cycles (Fig. 5h), respectively. The small volume expansion of the proposed Si/SiO_*x*_ nanofoils electrode is also another strength from a practical viewpoint^51,52^. From the corresponding TEM images and schematic illustration (Fig. 5i–m) of 2D Si/SiO_*x*_ nanofoils collected from the cycled electrodes, we confirmed that its unique morphology was still maintained without significant pulverization, even after 50 cycles, which is responsible for the superior electrochemical performance of the 2D Si/SiO_*x*_ nanofoils (Figs S7 and S8).

## Discussion

We have demonstrated a scalable and cost-effective synthesis of Si nanocrystals embedded 2D SiO_*x*_ ultrathin nanofoils with a thickness of about 8 nm, as well as centimeter scale large areas, *via* a solution-evaporation-induced interfacial sol-gel reaction of hydrogen silsesquioxane. Taking advantages of the 2D nanostructure, superior rate capability, and stable cycling performance of 2D Si/SiO_*x*_ nanofoils could yield a promising anode material for LIBs. More importantly, the excellent dimensional stability of 2D Si/SiO_*x*_ nanofoil electrode is comparable to that of commercial graphite anode, which offers new opportunities for developing next-generation LIBs. We also firmly believe that the synthetic strategy reported here, i.e., evaporation-induced interfacial sol-gel reaction, can open up a new avenue to prepare other inorganic 2D nanomaterials in a scalable and facile manner for various applications.

## Methods

### Material Preparation

To prepare 2D Si/SiO_*x*_ nanofoils, 1.0 ml trichlorosilane [HSiCl_3_, Aldrich, 99.8%] was centered in the middle of glass cylinder container(diameter: 14 cm) filled with 200 ml deionized water below 10 °C. After 20 min, 0.1 g of cracked-ice-like hydrogen silsesquioxane (HSQ, HSiO_1.5_) was obtained on the surface of the deionized water. The particles were carefully filtered and washed repeatedly. The filtered powders were gathered and dried in a vacuum oven at 110 °C. The 2D Si/SiO_*x*_ nanofoils were obtained by heat treatment at various temperatures from 700 to 1200 °C for 1 h under a 4% H_2_/Ar atmosphere with a heating rate of 20 °C min^−1^ and a flow rate of 0.5 L min^−1^.

### Structure Characterizations

The morphology and microstructure of the as-prepared 2D Si/SiO_*x*_ nanofoils were characterized by field-emission scanning electronic microscopy (FESEM; JEOL JSM-7000F), high-resolution transmission electron microscopy (HRTEM; JEOL 2100 F), atomic force microscopy (AFM; Park systems XE-100) and surface area and pore size analyzer (BET; Micromeritics Instrument Corporation 3Flex). For further inspection of the microstructure of the 2D Si/SiO_*x*_ nanofoils, powder X-ray diffraction (XRD) patterns were obtained by using a X-ray diffractometer (Empyrean, PANanalytical) equipped with a three-dimensional (3D) pixel semiconductor detector using Cu Kα radiation (λ = 1.54056 Å). The chemical states of the 2D Si/SiO_*x*_ nanofoils were further investigated by using a X-ray photoelectron spectrometer (XPS; Thermo Scientific Sigma Probe).

### Electrochemical Measurements

The electrodes were prepared by coating slurries consisting of the 2D Si/SiO_*x*_ nanofoils (70 wt%), a conducting agent (Super-P, 10 wt%), and polyacrylic acid binder (PAA, 20 wt%) dissolved in deionized water on Cu foil with a mass loading of 1.5 mg cm^−2^. The electrodes were dried at 120 °C for 12 h in a vacuum oven and were pressed under a pressure of 200 kg cm^−2^. The electrochemical performance was examined by assembling CR2032 coin-type half cells in an Ar-filled glove box. These cells were assembled with a polyethylene (PE) membrane as the separator and Li metal as a counter and reference electrode. 1 M LiPF_6_ dissolved in a mixed solvent of ethylene carbonate (EC) and diethyl carbonate (DEC) (3:7 v/v, Panax Etec Co. Ltd.) was employed as an electrolyte. The cells were tested in constant current–constant voltage (CC–CV) mode in a voltage window of 0.01–2.0 V *vs*. Li/Li^+^ at room temperature and at the high temperature of 60 °C. To prepare pre-lithiated 2D Si/SiO_*x*_ nanofoil anodes, in an Ar-filled glove box, the electrodes were firstly contacted with Li metal between two sheets of glass in a vessel filled with an electrolyte solution. Subsequently, pressure was introduced to the glasses directly. The electrodes were kept for different time (1, 3, and 6 h). In order to test the electrodes for the electrochemical performance, CR2032 coin-type half cells were assembled.

## Electronic supplementary material

Supplementary Information
