# Supplementary material for: Si Nanocrystal-Embedded SiOx nanofoils: Two-Dimensional Nanotechnology-Enabled High Performance Li Storage Materials
Source: Sci Rep. 2018 May 2;8:6904. doi: 10.1038/s41598-018-25159-4 (PMC5932046; doi:10.1038/s41598-018-25159-4)
Supplement: Supplementary file 1 — Supplementary Information [file 41598_2018_25159_MOESM1_ESM.docx]

Supporting Information for

**Si Nanocrystal-Embedded SiO*_x_* nanofoils: Two-Dimensional Nanotechnology-Enabled High Performance Li Storage Material**

**Hyundong Yoo^1,+^ , Eunjun Park^1,+^, Juhye Bae^1^, Jaewoo Lee^2^, Dong Jae Chung^1^, Yong Nam Jo^3^, Min-Sik Park^4,*^, Jung Ho Kim^2^, Shi Xue Dou^2^, Young-Jun Kim^5^, Hansu Kim^1,*^**

^1^ Department of Energy Engineering, Hanyang University, 222 Wangsimni-ro, Seongdong-gu, Seoul 133-791, Republic of Korea

^2^ Institute for Superconducting and Electronic Materials (ISEM), Australian Institute for Innovative Materials (AIIM), University of Wollongong, North Wollongong, New South Wales 2500, Australia

^3^ Advanced Batteries Research Center, Korea Electronics Technology Institute, Seongnam 463-816, Republic of Korea

^4^ Department of Advanced Materials Engineering for Information and Electronics, Kyung Hee University, Yongin 17104, Republic of Korea

^5^ SKKU Advanced Institute of Nanotechnology (SAINT), Sungkyunkwan University, Suwon 16419, Republic of Korea

^*^ Corresponding authors: mspark@khu.ac.kr; khansu@hanyang.ac.kr

^+^These authors contributed equally.

***Contents of supporting information***

**Figure S1.** Vapour pressure curves of trichlorosilane (TCS), silicon tetrachloride (STC), tetrahydrofuran (THF), ethanol (EtOH), and H_2_O. As shown in the comparisons of vapour pressure, TCS can be easily evaporated below 30 ^o^C compared to typical organic solvents and water.

**Figure S2.** Photographs of collected two-dimensional (2D) hydrogensilsesquioxane (HSQ) nanofoils. 2D HSQ nanofoils were obtained by sol-gel reaction of TCS in the home-made reactor at room temperature under ambient pressure. The size of resulting 2D HSQ nanofoils is about 5 cm X 5 cm.

**Figure S3.** (a) Low magnification field emission scanning electron microscope (FESEM) image of 2D Si/SiO*_x_* nanofoils, (b) high-magnification FESEM image of 2D Si/SiO*_x_* nanofoils, and (c) 3D TEM tomography image of 2D Si/SiO*_x_* nanofoils. Scale bar, (a) 5 μm and (b) 500 nm.

**Figure S4.** XPS spectra of 2D Si/SiO*_x_* nanofoils prepared at 1000 ^o^C; (a) Si 2p, (b) O 1s, (c) C 1s. The Si 2p spectrum indicates various intermediate oxidation states of Si after deconvolution with C1s excitation at 284.5 eV.

**Figure S5.** (a) Nitrogen adsorption and desorption isotherms and (b) pore-size distribution of Si/SiO*_x_* nanospheres and 2D Si/SiO*_x_*.

**Figure S6.** (a) Cycling performances of 2D Si/SiO*_x_* nanofoil electrodes (700 ^o^C, 800 ^o^C) at a constant current of 0.2 C (200 mA g^-1^) for 200 cycles. Galvanostatic voltage profiles of 2D Si/SiO*_x_* nanofoils prepared at various temperatures at a constant current of 0.2 C (200 mA g^-1^): (b) 900 ^o^C, 1000 ^o^C, and 1100 ^o^C, and (c) 700 ^o^C and 800 ^o^C. The initial reversible capacities of the anodes were 351.9 mAh g^-1^ (2D-700), 670.9 mAh g^-1^ (2D-800), 457.5 mAh g^-1^ (2D-900), 596.4 mAh g^-1^ (2D-1000), and 486.3 mAh g^-1^ (2D-1100) with initial coulombic efficiencies (IE) of 20.2%, 32.8%, 24.5%, 30.0%, and 29.9%, respectively.

**Figure S7.** Electrochemical performance of 2D Si/SiO*_x_* nanofoil (1000 ^o^C) electrodes at a constant current 0.2 C (200 mA g^-1^) at room temperature and high temperature (60 ^o^C): (a) galvanostatic voltage profiles and (b) cycling performance for 100 cycles.

**Figure S8.** Comparison of TEM images of 2D Si/SiO*_x_* nanofoils (1000 ^o^C) collected after the different cycles: (a) pristine, (b) after 1 cycle, (c) after 20 cycles, (d) after 50 cycles..

**Figure S9.** Comparison of HRTEM images of 2D Si/SiO*_x_* nanofoils (1000 ^o^C) collected after the different cycles: (a) pristine electrode, (b) after 1 cycle, (c) after 20 cycles, (d) after 50 cycles. Si nanocrystals embedded in the SiO*_x_* matrix were changed to amorphous structure after cycling.


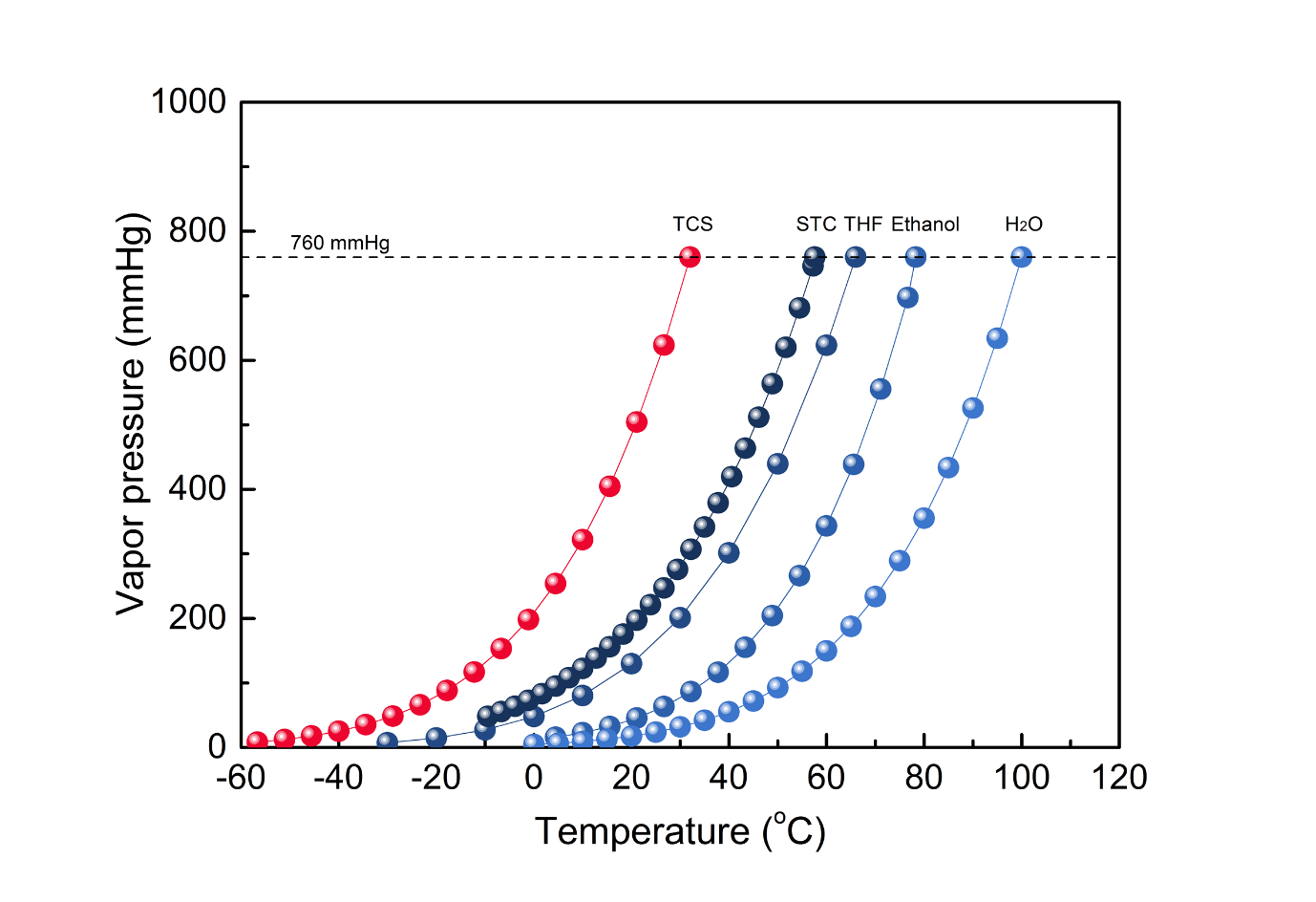


**Figure S1.** Vapour pressure curves of trichlorosilane (TCS), silicon tetrachloride (STC), tetrahydrofuran (THF), ethanol (EtOH), and H_2_O. As shown in the comparisons of vapour pressure, TCS can be easily evaporated below 30 ^o^C compared to typical organic solvents and water.


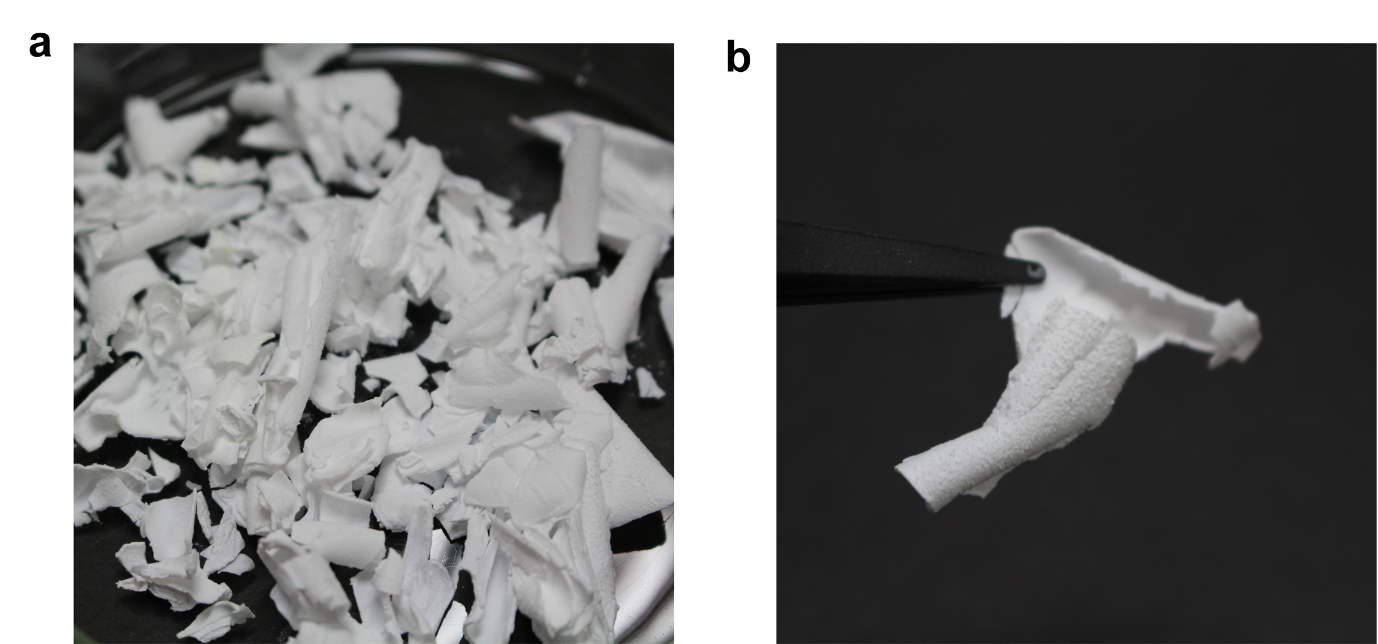


**Figure S2.** Photographs of collected two-dimensional (2D) hydrogensilsesquioxane (HSQ) nanofoils. 2D HSQ nanofoils were obtained by sol-gel reaction of TCS in the home-made reactor at room temperature under ambient pressure. The size of resulting 2D HSQ nanofoils is about 5 cm X 5 cm.


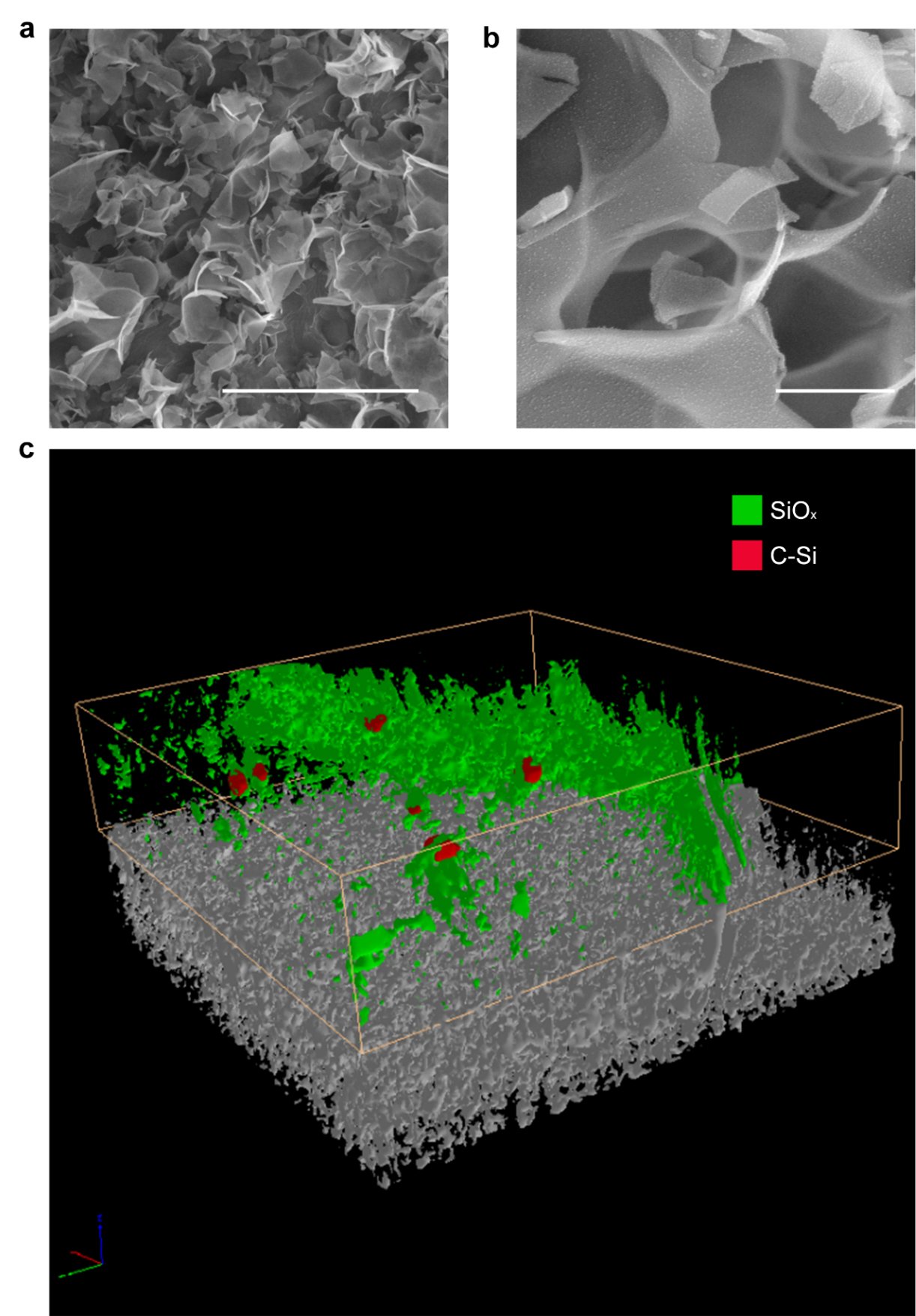


**Figure S3.** (a) Low magnification field emission scanning electron microscope (FESEM) image of 2D Si/SiO*_x_* nanofoils, (b) high-magnification FESEM image of 2D Si/SiO*_x_* nanofoils, and (c) 3D TEM tomography image of 2D Si/SiO*_x_* nanofoils. Scale bar, (a) 5 μm and (b) 500 nm.


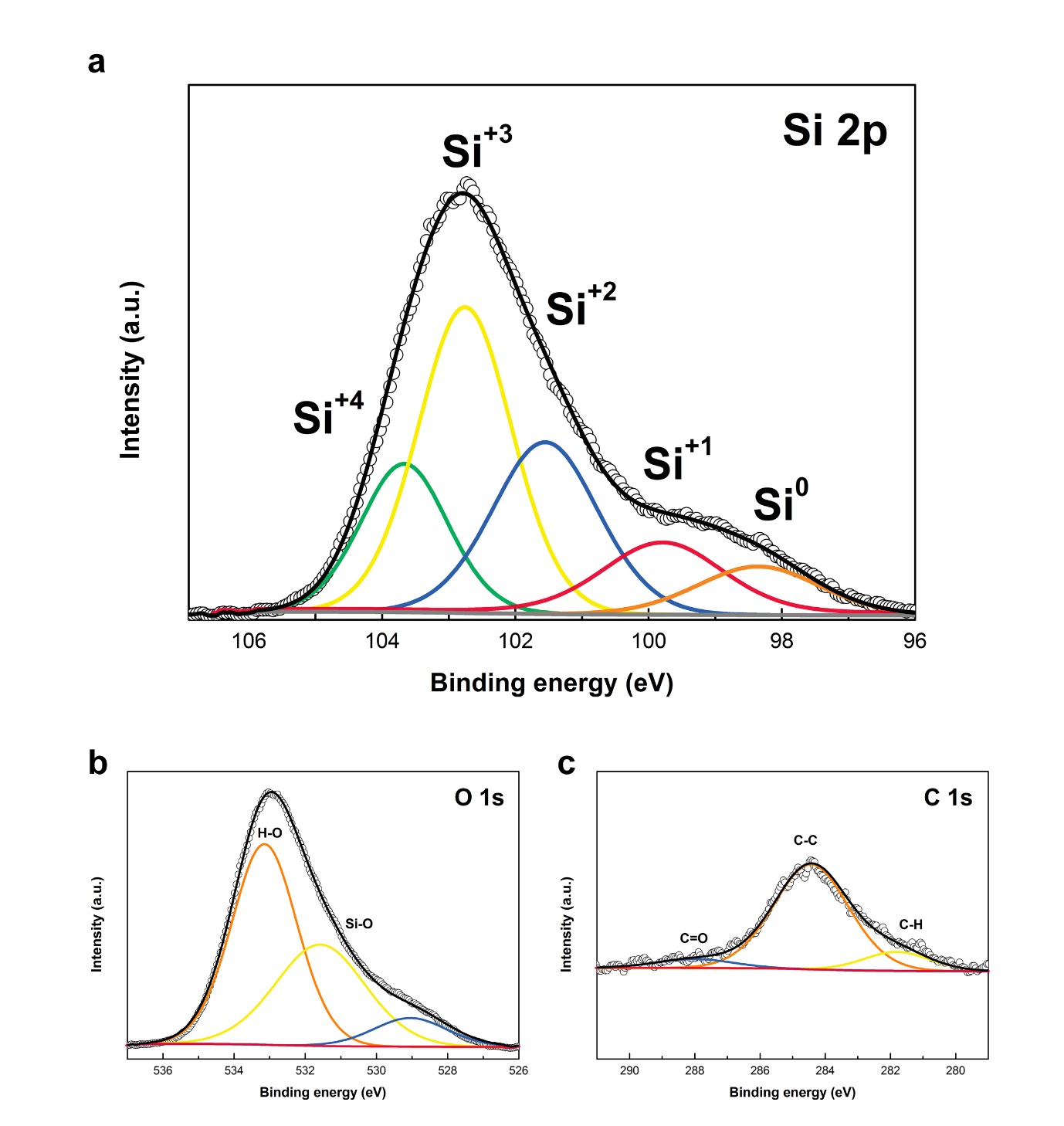


**Figure S4.** XPS spectra of 2D Si/SiO*_x_* nanofoils prepared at 1000 ^o^C; (a) Si 2p, (b) O 1s, (c) C 1s. The Si 2p spectrum indicates various intermediate oxidation states of Si after deconvolution with C1s excitation at 284.5 eV.

**Figure S5.** (a) Nitrogen adsorption and desorption isotherms and (b) pore-size distribution of Si/SiO*_x_* nanospheres and 2D Si/SiO*_x_*.


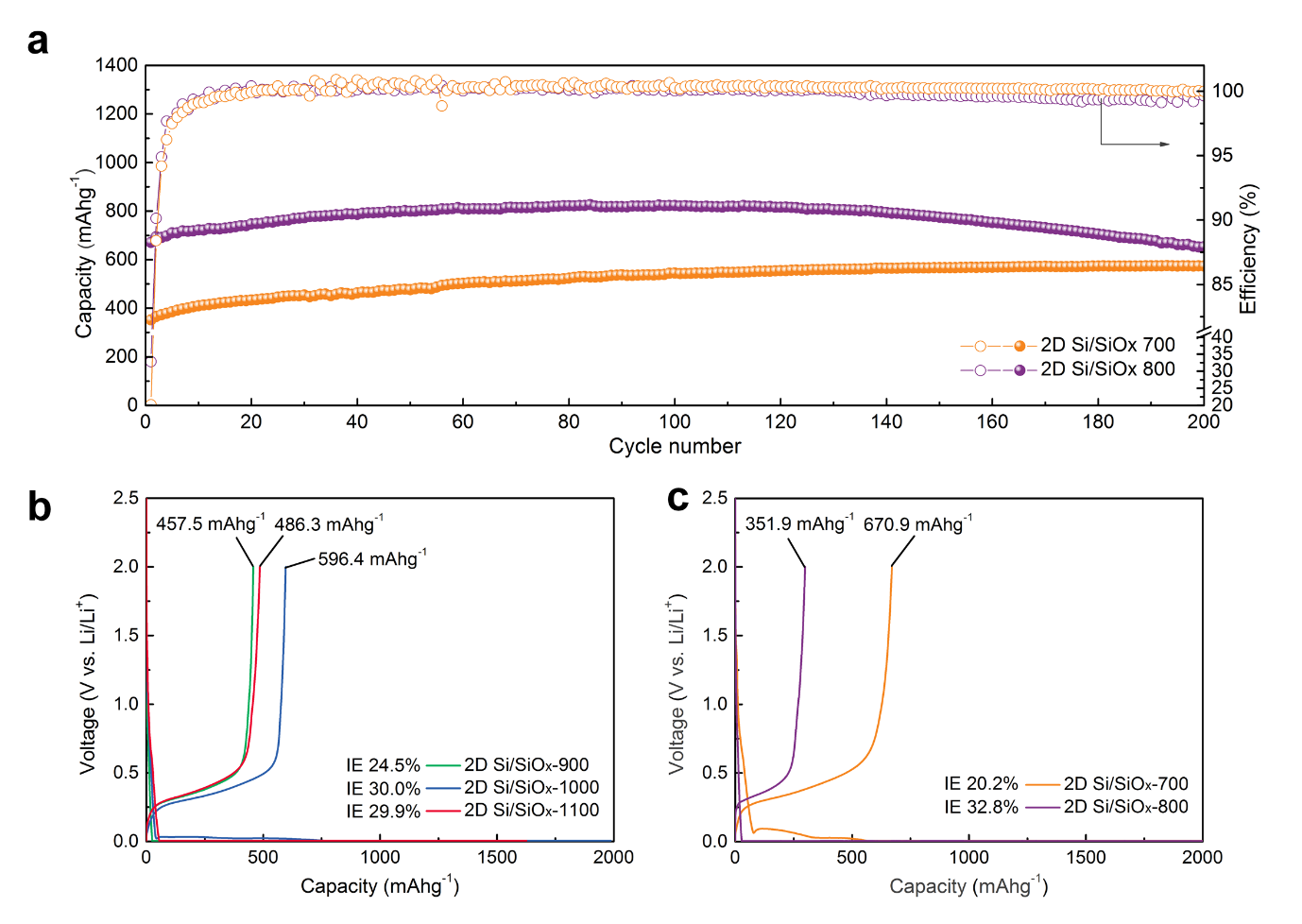


**Figure S6.** (a) Cycling performances of 2D Si/SiO*_x_* nanofoil electrodes (700 ^o^C, 800 ^o^C) at a constant current of 0.2 C (200 mA g^-1^) for 200 cycles. Galvanostatic voltage profiles of 2D Si/SiO*_x_* nanofoils prepared at various temperatures at a constant current of 0.2 C (200 mA g^-1^): (b) 900 ^o^C, 1000 ^o^C, and 1100 ^o^C, and (c) 700 ^o^C and 800 ^o^C. The initial reversible capacities of the anodes were 351.9 mAh g^-1^ (2D-700), 670.9 mAh g^-1^ (2D-800), 457.5 mAh g^-1^ (2D-900), 596.4 mAh g^-1^ (2D-1000), and 486.3 mAh g^-1^ (2D-1100) with initial coulombic efficiencies (IE) of 20.2%, 32.8%, 24.5%, 30.0%, and 29.9%, respectively.


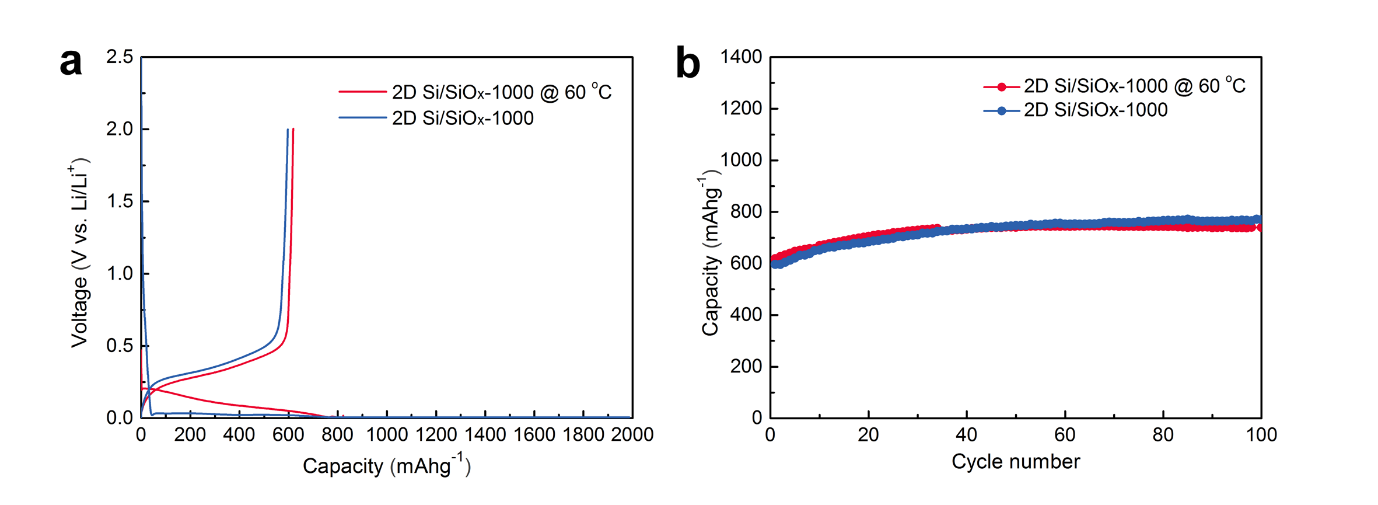


**Figure S7.** Electrochemical performance of 2D Si/SiO*_x_* nanofoil (1000 ^o^C) electrodes at a constant current 0.2 C (200 mA g^-1^) at room temperature and high temperature (60 ^o^C): (a) galvanostatic voltage profiles and (b) cycling performance for 100 cycles.


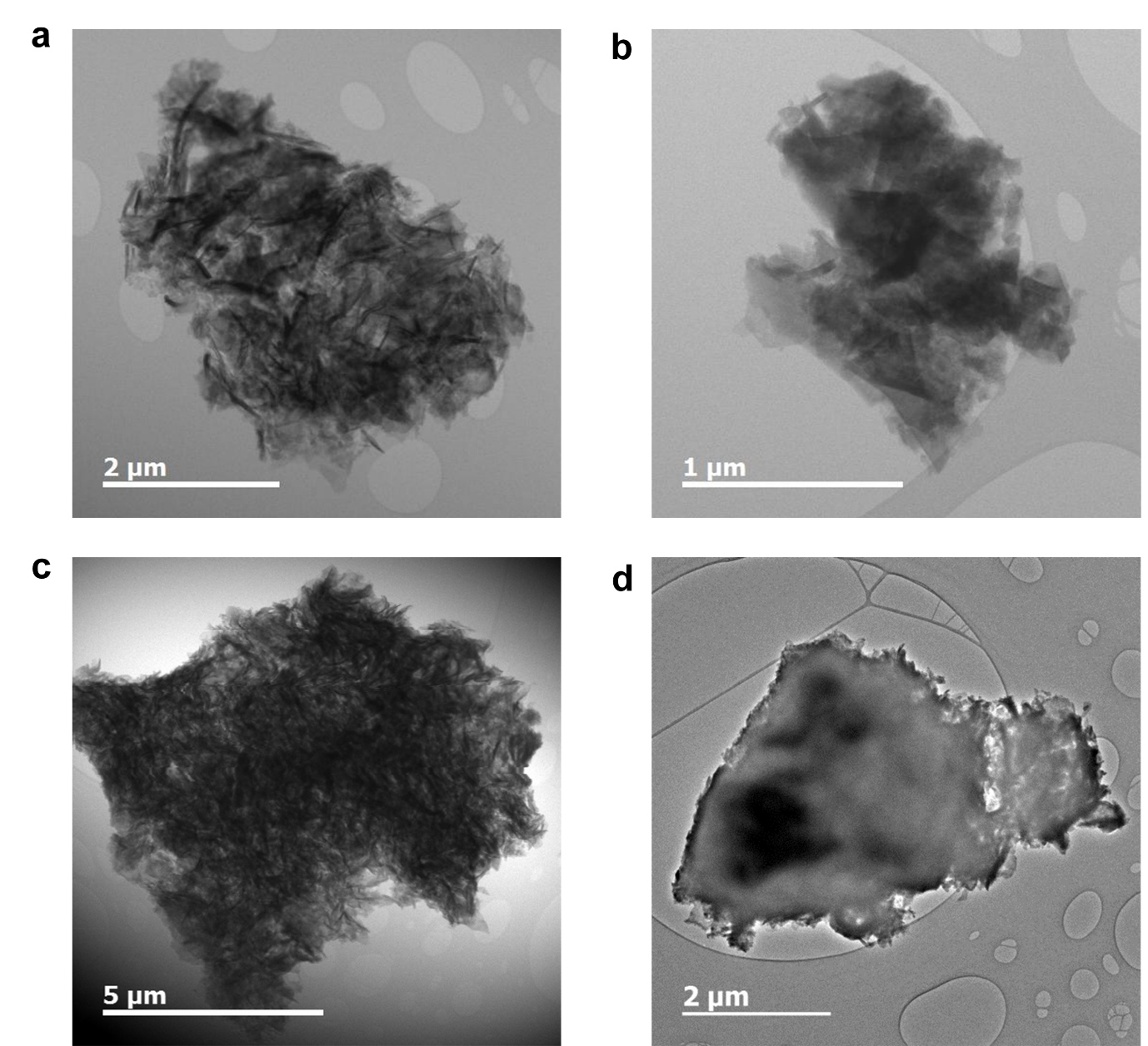


**Figure S8.** Comparison of TEM images of 2D Si/SiO*_x_* nanofoils (1000 ^o^C) collected after the different cycles: (a) pristine, (b) after 1 cycle, (c) after 20 cycles, (d) after 50 cycles.


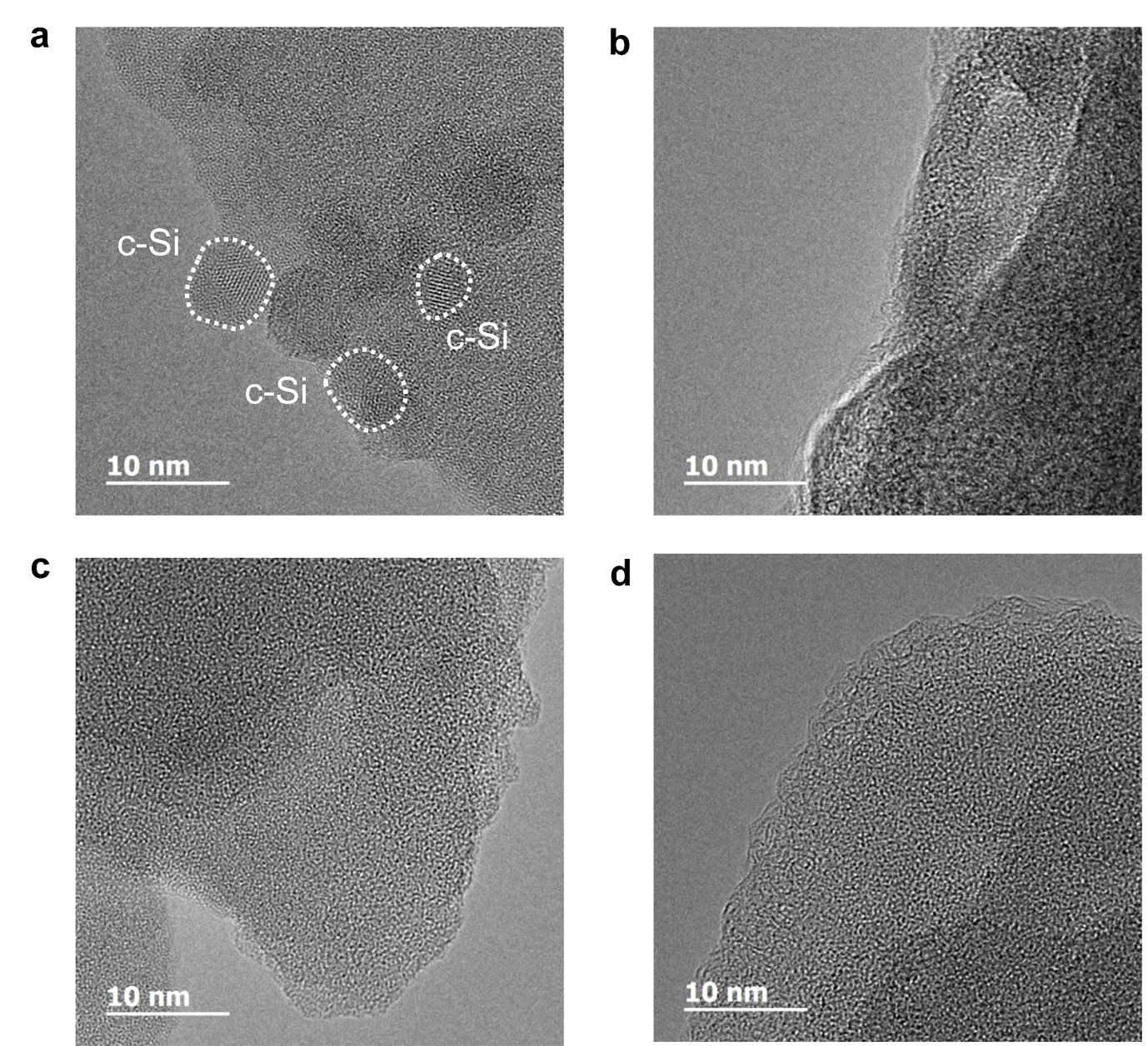


**Figure S9.** Comparison of HRTEM images of 2D Si/SiO*_x_* nanosheets (1000 ^o^C) collected after the different cycles: (a) pristine electrode, (b) after 1 cycle, (c) after 20 cycles, (d) after 50 cycles. Si nanocrystals embedded in the SiO*_x_* matrix were changed to amorphous structure after cycling.
